# Restricted Gene Flow among Lineages of *Thrips tabaci* Supports Genetic Divergence Among Cryptic Species Groups

**DOI:** 10.1371/journal.pone.0163882

**Published:** 2016-09-30

**Authors:** Alana L. Jacobson, Brian A. Nault, Edward L. Vargo, George G. Kennedy

**Affiliations:** 1 Department of Entomology, North Carolina State University, Raleigh, North Carolina, United States of America; 2 Department of Entomology, Cornell University, Geneva, New York, United States of America; Leibniz-Institute of Freshwater Ecology and Inland Fisheries, GERMANY

## Abstract

Knowledge of the relative influence of population- versus species-level genetic variation is important to understand patterns of phenotypic variation and ecological relationships that exist among and within morphologically indistinguishable cryptic species and subspecies. In the case of cryptic species groups that are pests, such knowledge is also essential for devising effective population management strategies. The globally important crop pest *Thrips tabaci* is a taxonomically difficult group of putatively cryptic species. This study examines population genetic structure of *T*. *tabaci* and reproductive isolation among lineages of this species complex using microsatellite markers and mitochondrial COI sequences. Overall, genetic structure supports *T*. *tabaci* as a cryptic species complex, although limited interbreeding occurs between different clonal groups from the same lineage as well as between individuals from different lineages. These results also provide evidence that thelytoky and arrhenotoky are not fixed phenotypes among members of different *T*. *tabaci* lineages that have been generally associated with either reproductive mode. Possible biological and ecological factors contributing to these observations are discussed.

## Introduction

Onion thrips, *Thrips tabaci* Lindeman (Thysanoptera: Thripidae), is a globally important insect pest that exhibits remarkable variation in biological and ecological traits within and among populations. Observed population differences include reproductive mode variation, ploidy, host plant preferences as well as differences in vector competency to transmit plant viruses and insecticide resistance [1–11]. Studies conducted over the last 20 years have produced phenotypic and genetic data that provide support for *T*. *tabaci* as a cryptic species complex comprised of three genetically distinct lineages exhibiting sufficient mitochondrial COI sequence variation to suggest that these lineages represent different species [1]. This genetic evidence is supported only by reported phenotypic differences in reproductive mode associations, host plant preferences, and differences in competency to transmit *Tomato spotted wilt virus* (TSWV). These lineages were characterized by Brunner et al. 2004 [1] and are generally defined as: Lineage 1—‘Arrhenotokous Tobacco Group’: a tobacco feeding lineage that has only been reported in Eastern Europe, is an efficient vector of TSWV, and exhibits arrhenotokous parthenogenesis (haploid males produced asexually, diploid females produced through sexual reproduction); Lineage 2 –‘Arrhenotokous Leek Group’: a leek/onion associated lineage that has been reported in Europe, Japan and the United States but is uncommon, is a competent vector of TSWV, and exhibits arrhenotokous parthenogenesis; and Lineage 3- ‘Thelytokous Leek Group’: a leek/onion associated lineage that has been reported worldwide and can be very abundant in the landscape, exhibits inter-clonal variation in its competence as a vector of TSWV, and exhibits thelytokous parthenogenesis (asexual populations comprised entirely of females).

Although classifications based on reproductive mode associations are generally accepted in the literature, population genetic studies to date have not resolved whether these groups are reproductively isolated from each other [6]. The first report in the literature suggesting their reproductive isolation involved a personal observation, rather than a comprehensive study, and stated that males from lineage 2 did not mate with females from lineage 3 [12]. More recent studies have highlighted the need to better define phylogenetic and population genetic relationships among these lineages. The first is a report by Sogo et al. [8] that questioned phylogenetic associations of the leek associated lineages 2 and 3 based on two female thrips that were classified as arrhenotokous because they produced male offspring but exhibited a lineage 3 COI haplotype. Although the authors concluded that these thrips represented a new lineage within lineage 3, they did not address the possibility that sex determination of offspring in *T*. *tabaci* is not a fixed phenotype within the lineage, as reported by several other authors [5,13,14]. Experiments conducted by Nault et al. [14] reported unmated females that had been individually reared to adult in isolation from each other produced both male and female offspring. Also, despite using similar rearing procedures to establish clonal lines from single females reared and maintained in isolation, Jacobson et al. [5] found that several lines from lineage 2 that produced males initially, stopped producing males (i.e. became phenotypically thelytokous) after one generation in the laboratory. Due to the carefully controlled nature of these experiments and the care that was taken in selecting and handling the thrips, it is extremely unlikely that these results were an artifact of contamination; moreover, multiple individuals from both of these studies exhibited these behaviors. The phenotypic variation in parthenogenesis and sex of offspring in this species has never been formally studied and has been largely ignored in the literature. Another recently published study conducted in New York, USA also challenges the strict association of *T*. *tabaci* lineages with reproductive mode [15]. In this study males from lineage 2 readily mated with females from lineage 3 in the laboratory, and examination of offspring from these matings showed that genetic exchange between parents occurred in 4 out of 75 offspring produced from mated females. It remains unknown whether or not interbreeding between members of different lineages occurs in the field [6,16].

Taken together the previously described studies highlight the need to better understand the reproductive isolation of individuals from different lineages, and characterize which lineages exhibit variation in sex determination of offspring by parthenogentically reproducing females. In our study we investigated population genetic structure of *T*. *tabaci* in New York onion agroecosystems using both COI sequence data and microsatellite markers. These agroecosystems were considered ideal for addressing our questions because past studies have reported the presence of individuals from lineages 2 and 3 in multiple locations throughout the state. The objectives of this study were to examine 1) population structuring in relation to geographic distance between onion production systems that are distributed throughout the state; 2) reproductive isolation of lineages 2 and 3 in this landscape; and 3) include individuals that were characterized in Nault et al.’s study [17] to examine the phylogenetic placement and population genetic structure of females asexually producing mixed-sex offspring.

## Materials and Methods

### Ethics Statement

No specific permits were required for the described field studies. Verbal permission was obtained from all private landowners to collect insects.

### Ecosystem

**Fig 1:**
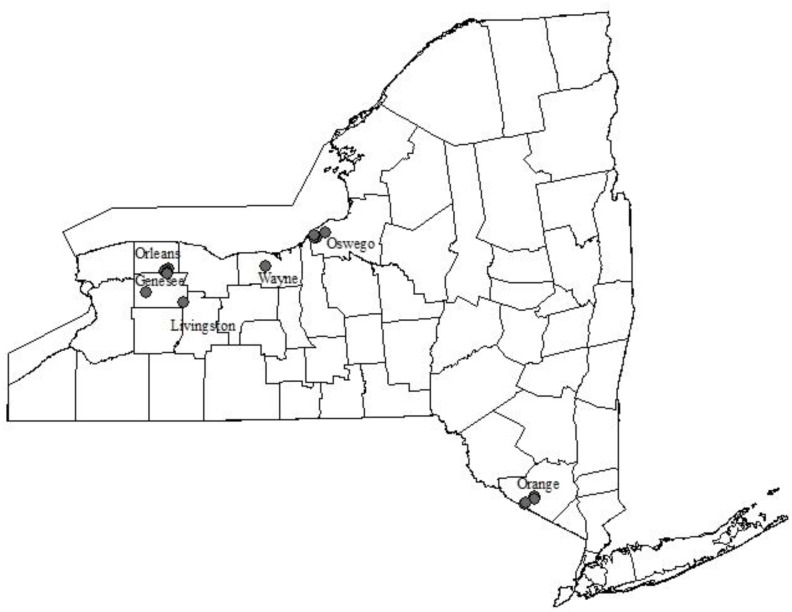
Map of New York collection locations for *Thrips tabaci*.

Populations of *T*. *tabaci* were sampled in discrete locations in New York, USA that were former lakes or swamps that had been drained for agricultural production over 100 years ago (Fig 1). These locations, which are characterized by rich, high organic soils and referred to as “mucks”, are currently used to produce a variety of crops, especially onion, *Allium cepa* L. Mucks are virtual islands in the New York landscape, separated by a mosaic of urban, forested and agricultural areas. The size, diversity and composition of the landscape surrounding these mucks differ among regions in New York. For example, the largest muck region in western New York (Orleans and Genesee Counties) exceeds 3,000 hectares and is surrounded by a landscape comprising primarily field and vegetable crops, but which also includes non-cultivated hosts for *T*. *tabaci* populations [18]. In central New York (Wayne and Oswego Counties), the landscape becomes less diverse and mucks tend to be smaller in size (40+ hectares), but much more numerous. The landscape surrounding these smaller mucks comprises mostly forested areas, generally considered to be poor habitat for *T*. *tabaci* due to the general lack of suitable host plants [18]. In southeastern New York (Orange County), the largest contiguous muck (approximately 14,000 ha) is surrounded and infused by suburban and natural areas, multiple vegetable and field crops, and commercial turf production. New York mucks are separated by straight-line distances of as little as 3.2 and as great as 400 km. Although *T*. *tabaci* adults likely disperse between onion fields and surrounding habitat patches [18], and between neighboring field and vegetable crop hosts [19,20] in western NY, the frequency of dispersal, distances traveled, and survival of *T*. *tabaci* dispersers within and among muck regions are not known. Although *T*. *tabaci* adults are capable of long-distance dispersal [21], populations are thought to be isolated because the mucks are considered to be “closed” systems due to their geographic isolation and the localized nature of past insecticide resistance problems [22,23].

### Insects

All *T*. *tabaci* were collected from onion plants grown in commercial production mucks in New York. In 2003, *T*. *tabaci* were characterized for their reproductive mode phenotype (see [14]). Briefly, larvae were collected from a total of 10 onion fields; two different fields in each of five counties (Fig 1, S1 Table). After collection, immature thrips were reared separately to adult in individual Petri dishes to ensure adult females would be unmated. The resulting virgin, adult females were then allowed to reproduce asexually on cabbage leaf discs. Their offspring were reared to adult and their sex identified to classify the reproductive mode of their mother; virgin females producing female offspring were phenotyped as thelytokous, virgin females producing males were phenotyped as arrhenotokous, and virgin females producing both sexes were phenotyped as deuterotokous [14]. The sex of thrips was determined visually under a compound light microscope and recorded for each individual. Individuals with arrhenotokous phenotypes were collected from at least one field in each county (a total of five fields), individuals with thelytokous phenotypes were collected from each field, and individuals with deuterotokous phenotypes were collected from four fields in which thelytokous and arrhenotokous individuals were also found. The cohorts of offspring from these experiments were stored separately in 95% ethanol at -20°C. A single offspring from each cohort, produced from a virgin female, was subjected to our genetic analyses (8–13 individual thrips per location).

In 2011 and 2012, *T*. *tabaci* were collected from onion fields in eight different mucks from five counties, both early- (May and June) and late-season (July and August) (S1 Table). Early- and late-season sampling was done to increase our chances of collecting arrhenotokous individuals, and to eliminate bias in population analyses that may result from different genotypes being eliminated from the landscape due to insecticide use during the season. Early-season samples were taken when adults were beginning to colonize young onion plants, before males have been observed in populations [14], and before insecticides were applied to control thrips infestations. Late-season samples were taken after 2–3 generations of *T*. *tabaci* had developed in the same fields, and at a time when males have been previously reported to occur in some of the locations sampled [14]. Samples may be missing for some locations because the fields had not been colonized at the time early-season collections were made, or had been harvested prior to collection of late-season samples. Only one adult was collected per plant, and plants were sampled randomly across the onion fields to avoid collecting multiple offspring from the same female. At least 10 individuals from each county at each collection date in 2011 and 2012 were included in the genetic analyses. We did not conduct rearing experiments to identify the reproductive mode of the adults in the 2011–2012 samples. Instead, we identified lineage membership of field-collected individuals by analyzing COI sequences [1,6,9] (see below).

### DNA processing and genotyping

Genomic DNA was extracted from individual thrips using the DNeasy Kit (Qiagen, Valencia, CA). Microsatellite and COI markers were amplified according to the protocol in Jacobson et al. [6,7]. The COI markers were used to identify lineage membership of individuals collected from different locations in 2003 (N = 19), 2011 (N = 45), and 2012 (N = 60). For samples collected in 2003, individuals with arrhenotokous, deuterotokous and thelytokous reproductive mode phenotypes were included. In 2011 and 2012 a random sample of individuals from each field and collection date were chosen. Forward and reverse sequence alignments of COI DNA sequences were performed using the Vector NTI advanced^®^ software (Invitrogen, Carlsbad, CA) before analysis. Sequences were condensed into haplotypes using the Map module [24] in Mobyl SNAP Workbench [25]. Phylogenetic relationships were examined using Molecular Evolutionary Genetics Analysis (MEGA), Version 6 [26]. Maximum likelihood (ML) trees were constructed using the Jukes-Cantor [27] gamma distributed model in which all characters were equally weighted and 1,000 bootstrap replications were conducted. *Thrips hawaiiensis* Morgan (Genbank: AB277226), *T*. *palmi* Karney (Genbank: KF144144), *Frankliniella occidentalis* Pergande (Genbank; JX235930), and *F*. *intonosa* Trybom (Genbank: NC_021378) COI sequences from Genbank were included in the analysis as outgroups.

Genetic analyses of these populations were also conducted using nine microsatellite loci (20, 24, 27, 29, 33, 43, 47, 48, 49) described in Jacobson et al. [6]. For these analyses 122 individuals in 2003, 359 individuals in 2011 (200 collected early-season, 159 collected late-season), and 337 individuals in 2012 (166 collected early-season, 171 collected late-season) were included. Because *T*. *tabaci* has populations of mixed ploidy (diploid females and tetraploid females), and allele dosage in partial heterozygotes cannot be determined (due to insufficient DNA from a single thrips to conduct both ploidy and genetic analyses), microsatellite genotypes were analyzed using PCA cluster analysis executed in the R program Polysat [28]. This program is designed to handle samples of mixed ploidy, and estimate allele dosage in partial heterozygotes. Previously, maximum number of alleles per loci (calculated by Polysat) was shown to reflect diploidy or tetraploidy in *T*. *tabaci* (for more information about polyploidy in *T*. *tabaci* or relationships between number of alleles and ploidy see [7]). If the maximum number of alleles per locus was three or four, the individual was classified as tetraploid [6,7]. Individuals with a maximum of two alleles per locus were classified as diploid, and individuals with one allele per locus, haploid. Polysat was used to estimate allele copy number in partial heterozygotes to calculate pairwise genetic distances using the method described by Bruvo et al. [29], and perform a principal component analysis (PCA) to examine population structuring. PCA is a cluster analysis that partitions genetic variation among axes of differentiation. PCA can be used to examine population structure without *a priori* assumptions of population number or membership, or requiring usual assumptions of random mating, Hardy-Weinberg equilibrium and linkage equilibrium [30]. Analyses using PCA were conducted to examine population structuring separately in 2003, 2011, and 2012, as well as a combined analysis that included samples from all three years. *F*-statistics were also calculated in Polysat using the subsample of individuals whose COI haplotype was known (Table 1). Individuals were grouped into populations based on COI haplotype and on clustering identified in both PCA and STRUCTURE analyses.

**Table 1:** Pairwise *F*_ST_ between NY populations of *T*. *tabaci* collected in 2003, 2011, 2012 by COI Haplotype.

Pairwise *F*_ST_				
	NY-HT1 (L*3)	**NY-HT2 (L3)**	**NY-HT3 (L3)**	**NY-HA1 (L2)**
**NY-HT1 (L3)**	0.00			
**NY-HT2 (L3)**	0.04	0.00		
**NY-HT3 (L3)**	0.09	0.10	0.00	
**NY-HA1 (L2)**	0.16	0.20	0.16	0.00

* Lineage.

Bayesian model-based clustering of individuals into populations was examined using STRUCTURE version 2.3.4 [31] for individuals in 2003, and 2011–2012. Polysat was used to generate infiles for STRUCTURE that included estimated allele dosage for tetraploid individuals. Ten iterations for each value of K (1–10) were run under an admixture model assuming correlated allele frequencies using a burn-in period of 10,000 and 100,000 MCMC replications after burnin. STRUCTURE HARVESTER [32] was used to calculate K. Missing genomes in diploids and haploids were coded as missing (see STRUCTURE manual for more information). Individuals were only included if their COI haplotype was known.

## Results

### Linage identification using COI sequencing

**Fig 2:**
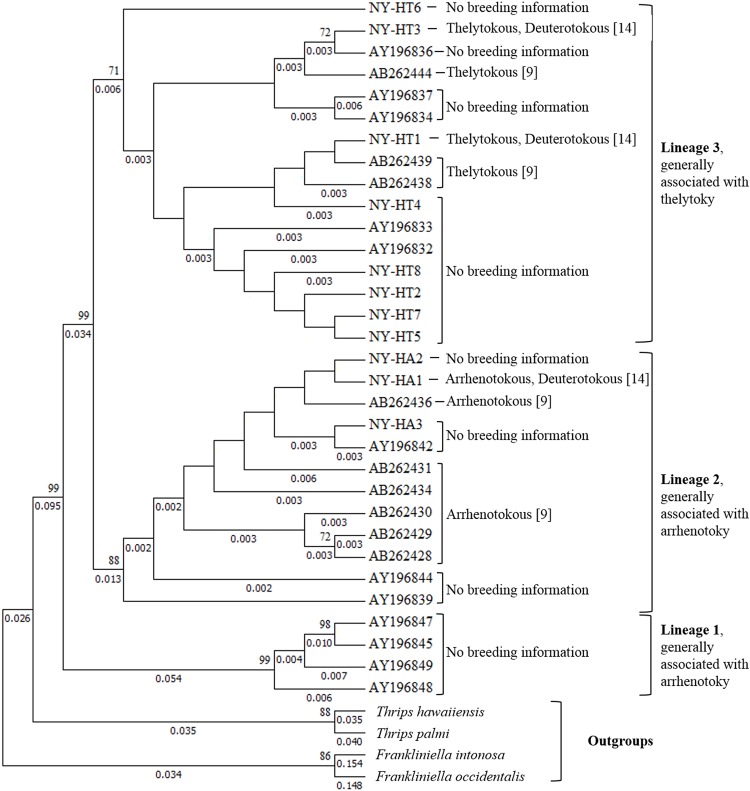
Molecular phylogenetic analysis by Maximum Likelihood method. The evolutionary history was inferred by using the Maximum Likelihood method based on the Jukes-Cantor model [27]. The tree with the highest log likelihood (-1420.3799) is shown. The percentage of trees in which the associated taxa clustered together is shown next to the branches. Initial tree(s) for the heuristic search were obtained automatically by applying Neighbor-Join and BioNJ algorithms to a matrix of pairwise distances estimated using the Maximum Composite Likelihood (MCL) approach, and then selecting the topology with superior log likelihood value. A discrete Gamma distribution was used to model evolutionary rate differences among sites (6 categories (+*G*, parameter = 0.5955)). The analysis involved 36 nucleotide sequences. There were a total of 313 positions in the final dataset. Evolutionary analyses were conducted in MEGA6 [26]. Bootstrap values above 70, and branch lengths above zero are displayed. Reproductive modes and lineages are labeled based on information from references [1, 9, 11, 14].

A 629 bp fragment of the COI was sequenced for a subset of *T*. *tabaci* individuals collected in all three years. Phylogenetic relationships examined using ML methods in MEGA identified two major clades in this study, which corresponded to lineages 2 and 3 (Fig 2) [1,6,7,9,17]. Most haplotypes collected in this study were previously reported in New York: lineage 3 haplotypes NY-HT1, NY-HT2/NY-HT5 (these two could not be distinguished based on the sequences used in this analysis), NY-HT3, NY-HT6 [17], and lineage 2 haplotype, here designated as NY-HA1 [15]. Two new haplotypes in lineage 2, NY-HA2 (Genbank: KR152257), and NY-HA3 (Genbank: KR152258) were identified in this study. Of the 178 individuals sequenced, only 16 belonged to lineage 3 (12 in 2003, three in 2011, and one in 2012).

An examination of the individuals collected in 2003 that came from cohorts of offspring from females phenotyped as deuterotokous in 2003 showed that females from both lineage 2 and lineage 3 can produce male and female offspring asexually (e.g., NY-HT1, NY-HT3 and NY-HA1). The cohorts of offspring from virgin females belonging to lineage 2 had sex ratios highly skewed toward male production (e.g., NY-HA1). Virgin females from 2003 from lineage 3 produced cohorts of offspring for which the opposite was observed; a greater proportion of their progeny were females (e.g., NY-HT1, NY-HT3) (see [14]).

### Population structuring and ploidy of New York *T*. *tabaci*

Mean allelic richness of microsatellite loci was calculated for COI haplotypes; all individuals from lineage 2 were grouped together, and different COI clonal types from lineage 3 were each analyzed separately. Maximum number of alleles per locus ranged from 3 to 15, and mean allelic richness was 6.5 alleles per locus across all individuals, 5 for lineage 2, and 6.5 for lineage 3. Among individuals with different COI haplotypes from lineage 3, mean allelic richness varied: 5.9 for NY-HT1, 9 for NY-HT2, and 5.2 for NY-HT3, respectively.

**Fig 3:**
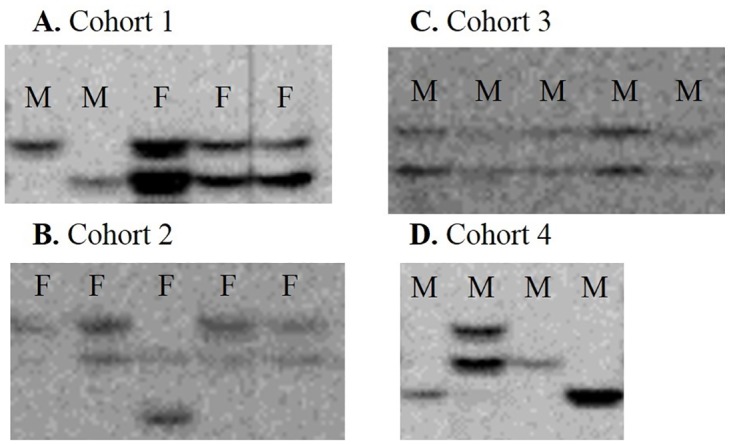
Gel images of microsatellites from diploid males. *Thrips tabaci* male (M) and female (F) siblings, produced though parthenogenetic reproduction, did not always share identical genotypes at the same loci (A, B, D), and diploid males were discovered (C, D).

Ploidy estimates based on maximum number of alleles per locus resulted in all of the females in 2003 being classified as tetraploid, and both haploid and diploid males were observed. Of 23 males examined in 2003, 16 were classified as haploid and seven, diploid. In 2011 and 2012, 43 females were classified as diploid, and the remaining 653 were classified as tetraploid. No males were collected in 2011 or 2012. Diploid males had never previously been reported for *T*. *tabaci;* therefore, these samples were re-run. As an additional check, DNA was extracted from a subsample of brothers from the cohorts in which putative diploid males were observed, and amplified at the same loci showing duplicate bands (Fig 3). When the male was produced from a virgin female that also produced females, multiple sisters from these cohorts were also included. Other brothers from these cohorts were observed to have two alleles/bands at the same loci. In addition, not all brothers and sisters shared identical genotypes at all loci in arrhenotokous and thelytokous individuals (Fig 3). Therefore, we concluded that some females are capable of producing diploid males and that offspring produced parthenogenetically are not all genetically identical. Reproductive cytology and sex determination systems in Thysanoptera are not well understood; however, these results show that thelytoky occurs via automixis since gamete duplication, mitotic thelytoky, and endoreplication would result in identical genotypes among siblings. The production of diploid males is consistent with tetraploidy in this species, provided that the reduction of the maternal chromosome number in a tetraploid female (similar to haplo-diploid mating systems) would produce a diploid male. More information on gamete formation in *T*. *tabaci* lineages, clonal groups, and individuals with different ploidies is needed to understand the observed relative frequency of haploid and diploid males and the mechanisms responsible for their production.

**Fig 4:**
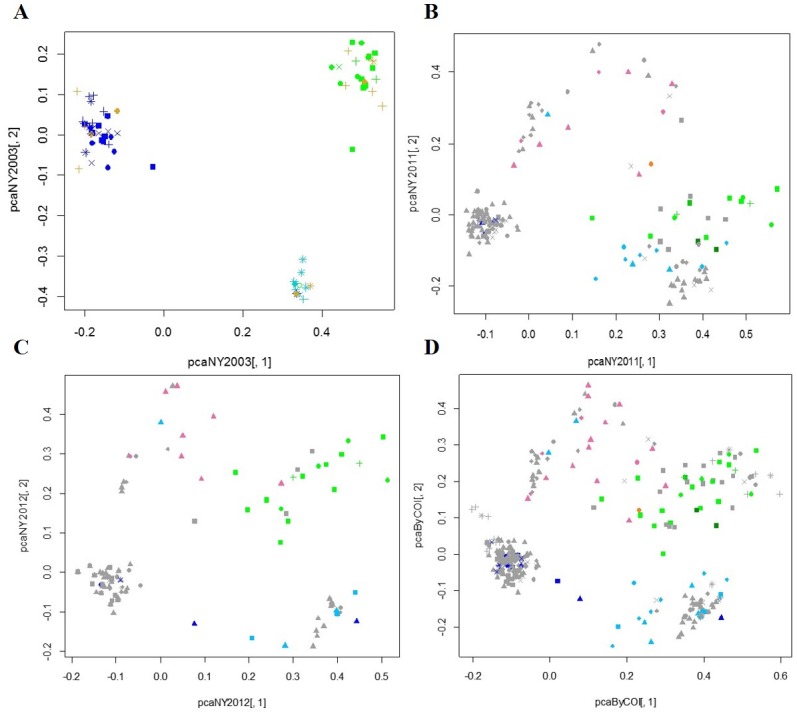
PCA of microsatellite markers from *Thrips tabaci* populations collected from New York. In 2003 (A), three genetically distinct populations are shown that represent individuals from COI haplotype NY-HT1 (dark blue), NY-HT3 (light blue), or NY-HA1 (green), and individuals phenotyped as deuterotokous (gold) are found in all three population clusters. Populations in 2011 (B) and 2012 (C) contain the aforementioned haplotypes and additional NY-HT2 (pink), NY-HT6 (orange), NY-HA2 and NY-HA3 (both dark green). (D) is a combined PCA with individuals from 2003, 2011 and 2012. Individuals in black were not characterized for COI haplotype. Symbols represent different collection locations: squares = Orange Co., circles = Oswego Co., triangles = Genesee Co., diamonds = Livingston Co., + = Yates Co., x = Wayne Co., * = Orleans Co.

The results from the PCA, STRUCTURE and *F*_ST_ analyses all show that these *T*. *tabaci* populations are genetically structured by COI lineage and clonal groups. The principal component analysis conducted on the 2003 samples previously characterized for reproductive mode based on sex of their offspring produced three distinct populations, each corresponding to one of the three COI haplotypes that were collected that year (Fig 4A); one comprised solely of individuals from lineage 2 (NY-HA1), and the other two comprised of individuals from lineage 3 (one cluster of individuals characterized as NY-HT1 and one cluster of individuals characterized as NY-HT3). There was no pattern to suggest geographic isolation of individuals collected from different locations using PCA, even when individuals from these populations were analyzed separately (data not shown). The cluster dominated by NY-HT1 (upper left) was comprised of individuals collected from nine of the 10 field locations from each county. The smaller cluster dominated by NY-HT3 (lower right) was comprised of individuals from six fields in four counties, and the cluster dominated by NY-HA1 (upper right) was comprised of individuals found from four fields in four counties (Fig 4A). This analysis suggests that individuals from lineages 2 and 3 do not interbreed in areas where they co-exist.

Results from PCAs conducted on individuals collected in 2011 and 2012 (Fig 4B and 4C, respectively) generally show populations with the same COI haplotype clustering together; however, there are additional clusters that include haplotypes not collected in 2003 and the delimitations between clusters are not as distinct as they were for 2003 populations. In 2011–2012 an additional two COI haplotypes were identified from lineage 3, NY-HT2 and NY-HT6, and each was collected from all five counties. An additional two COI haplotypes from lineage 2 were also identified, NY-HA2, and NY-HA3; both collected from Orange County. NY-HT2 appears as a separate group that is genetically distinct from the other COI haplotypes from lineage 3. Although populations are generally comprised of individuals with the same COI haplotype, individuals from different lineages and clonal groups cluster together, which suggests gene flow may occur among these different groups. An individual from the lineage 3 associated COI haplotype, NY-HT2, clusters with individuals from lineage 2. Additionally, individuals with different lineage 3 associated COI haplotypes in 2011 and 2012 group together; one individual NY-HT1 is found in the cluster of individuals with NY-HT3, and one individual with NY-HT3 clusters among individuals with NY-HT2. This could occur if rare males produced by females in lineage 3 (which has generally been presumed to be comprised exclusively of individuals with thelytokous phenotypes) are reproductively viable and occasionally mate with females in lineage 3. Similar to 2003, there was no evidence for geographic structuring among locations in 2011–2012, or when populations were analyzed separately (data not shown). There were also no major differences in population structuring between early- and late-collected individuals in 2011 or 2012 (data not shown).

**Fig 5:**
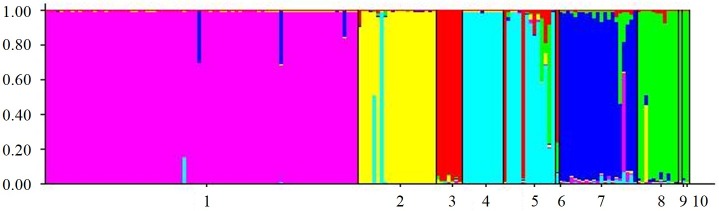
Graphical output of population membership identified using STRUCTURE. Populations labeled on x-axis are: 1) NY-HT1 collected in 2003; 2) NY-HT1 collected in 2011–2012; 3) NY-HT2 collected in 2011–2012; 4) NY-HT3 collected in 2003; 5) NY-HT3 collected in 2011–2012; 6) NY-HT6 collected in 2011–2012; 7) NY-HA1 collected in 2003; 8) NY-HA1 collected in 2011–2012; 9) NY-HA2 collected in 2011; 10) NY-HA3 collected in 2011–2012.

The results from STRUCTURE and STRUCTURE HARVESTER result in an optimum value of K = 2 populations. Although this result may be expected based on the presence of two distinct lineages identified in COI analyses, this grouping places individuals from lineages 1 and 2 into the same group (S1 Fig), which does not reflect genetic structuring of individuals from these lineages identified in the COI or PCA analyses. Other analyses run in STRUCTURE and STRUCTURE HARVESTER that analyzed individuals by year, lineage, and clonal group (data not shown), also returned optimal values of K = 2, which does not necessarily reflect the genetic structure identified in other analyses. Interpretation of K is not always straightforward, and the low sample sizes for some of the clonal groups and populations collected in this study may be biasing calculations to estimate K [33]. Among all of these analyses, however, there was some value of K for which individuals would generally be grouped by year and clonal group. The results presented here reflect the general population assignment of individuals among all of our analyses, and best corroborate the results obtained with the COI and PCA analyses. The results from STRUCTURE when K = 6 shows most individuals cluster together based on COI haplotype, and that individuals sharing the same COI haplotype can be further separated into distinct groups from 2003 and 2011–2012 (Fig 5 shows K = 6 output; figures for K = 2–10 provided in S1 Fig). The exceptions to this are the same individuals that clustered with different COI haplotypes described above and shown in the PCA. Little admixture is observed among individuals with different COI haplotypes, however, low levels occur within and among individuals from lineages 2 and 3. All lineage 2 individuals from 2011–2012 are grouped into the same population at this K value.

The *F*_ST_ values calculated between different clonal groups using Polysat show a larger degree of genetic differentiation between lineage 2 and 3 individuals than between different COI haplotypes within lineage 3 (Table 1). *F*_ST_ values calculated among individuals collected from different counties show no clear pattern of differentiation (data not shown).

## Discussion

### Ongoing gene-flow among diverging lineages

These results provide new evidence consistent with *T*. *tabaci* as a cryptic species complex even though ongoing gene flow occurs. Results from this study and Li et al. 2015 [15] show that gene-flow events are rare; however, it is not clear whether or not offspring produced from these events persist in the environment. Future studies examining both gene flow among different clonal groups of *T*. *tabaci*, and the fitness of their offspring to determine species-level classification of *T*. *tabaci* are needed. Both Bayesian and PCA analyses of microsatellite markers show populations are generally structured by clonal groups that can be differentiated based on COI sequences. This is consistent with previous studies reporting phenotypic differences in virus transmission and host plant associations among different clonal groups of *T*. *tabaci* [5,10], and indicates that clonal and population-level genetic differentiation likely plays an important role in phenotypic variation of economically important traits commonly studied. Bayesian analyses also demonstrate that the genetic diversity of clonal groups changes over time because microsatellite markers differentiated individuals collected in 2003 from individuals collected in 2011–2012, even when they shared the same COI haplotype. Interbreeding among clonal groups may be an important mechanism for increasing genetic diversity in *T*. *tabaci* that contributes to the success of individuals in lineage 3 (most abundant worldwide and generally associated with thelytoky). Patterns of population differentiation calculated using Polysat and STRUCTURE provide evidence for gene flow in the field, and support results from a recent laboratory investigation showing *T*. *tabaci* from lineages 2 and 3 successfully mate in the laboratory at low frequency [15]. Ongoing gene flow among diverging species and occasional sexual reproduction by asexual taxa have been reported for other insects [34–38], including between thelytokous females and males they spontaneously produce [39,40]. Occasional sexual reproduction has been implicated in increasing genetic diversity in asexual species, and in this study mean allelic richness was higher for *T*. *tabaci* from lineage 3 (generally associated with thelytokous parthenogenesis) than for lineage 2 individuals (generally associated with arrhenotokous parthenogenesis). Gene flow among divergent asexual and sexual taxa also provides opportunities for hybridization that can contribute to creation of new parthenogenetic lineages [39], ecological success of hybrids [41,42], and genome duplication events [43,44]. Future studies are needed to better understand factors driving the divergence of *T*. *tabaci* lineages, and the influence of ongoing gene flow on genetic diversity and polyploidy.

### Population genetic structure of *T*. *tabaci* in New York

Results from all of the population genetic analyses indicate that populations of *T*. *tabaci* in the New York landscape are structured according to clonal groups. Our data provide no evidence for geographic structuring of *T*. *tabaci* populations in New York, which contrasts with previous findings of genetic differentiation of *T*. *tabaci* in North Carolina [6,7]. The North Carolina system differed from the New York system in that the habitat for *T*. *tabaci* in North Carolina is neither temporally nor spatially stable; populations of *T*. *tabaci* are highly variable year-to-year and location-to-location, and populations rarely reach high numbers, which limits the number of individuals dispersing in the landscape [45]. In comparison, New York *T*. *tabaci* populations commonly reach densities of one or more thrips per onion plant in managed onion mucks of 40 to 14,000 hectares per muck and ca. 247,000 plants per hectare. With populations reaching such high levels in New York, there will be more individuals present in the landscape to disperse, and a greater likelihood that some dispersers successfully establish in other areas. Dispersing *T*. *tabaci* have been caught on traps using unmanned, aerial vehicles flown 50 to 60 m above onion fields in the Elba muck [21], suggesting a capacity for long-distance dispersal across this region that may have contributed to the absence of geographic structuring in New York. Other potential contributors include the common practice of importing onion transplants and bulbs from the southwestern US and Canada to be re-packaged and sold. These have been found to harbor *T*. *tabaci* (Nault unpublished) that may establish populations or mate with resident populations. However, to our knowledge, transplants have not been planted in some of the mucks sampled in our study.

Another notable result from the population genetic study is the apparent change in population structure between 2003 and 2011–2012, even among individuals from the same clonal groups. The PCA conducted on individuals in 2003 show that individuals from the three COI haplotypes collected that year cluster into three distinct groups, whereas in 2011–12 the genetic variation is greater within population clusters. This difference in genetic variation, within and among clusters suggests that something changed in this habitat that has allowed a greater amount of genetic variation to exist in these populations in 2011–2012 compared with 2003. During 2001–2003 widespread resistance to pyrethroid insecticides in *T*. *tabaci* was documented across onion production mucks in New York. This resistance was the result of intense selection imposed by the widespread, prophylactic use of these insecticides for *T*. *tabaci* management [22,23]. It is possible that this selection for pyrethroid resistant genotypes constrained genetic diversity present in *T*. *tabaci* populations within New York onion production systems during 2003. Currently, management programs for *T*. *tabaci* in New York onion fields include rotations of 3–4 insecticides, each with a different mode of action and, ideally, targeting different *T*. *tabaci* generations during the growing season. This change in insecticide stewardship, which is designed to delay the onset of resistance by reducing consistent selection pressure on *T*. *tabaci* populations may underlie the greater genetic diversity observed for the 2011–2012 populations. Further research would be needed to define the role of management practices on population genetic diversity.

### Reproductive mode and male production

Across the tree of life there is an impressive amount of diversity in reproductive behavior and sex determination mechanisms ([46] and references therein). Factors that regulate male production and the abundance of male-producing populations are not understood for members of Thysanoptera but merit discussion, given our observations on asexual production of male and female *T*. *tabaci* offspring in both lineage 2 and lineage 3. *T*. *tabaci* is one of the few thysanopterans reported to exhibit multiple reproductive mode phenotypes; arrhenotoky is the reproductive mode generally associated with this order [47], but there have been reports of thrips species that exhibit other reproductive modes or multiple reproductive modes [47–56]. The data from Nault et al. [14] combined with the population genetic data from 2003 in our study show that reproductive modes defined as either arrhenotokous or thelytokous in *T*. *tabaci* are phenotypes that may vary over time. Based on these observations, caution is needed when making conclusions about reproductive mode that are based on phenotypic classifications alone [8].

Evidence to date suggests that *T*. *tabaci* is a species complex that exhibits important biological, genetic, reproductive and phenotypic variation that needs to be carefully considered when designing future experiments. This study shows that genetic variation occurs at the species- population- and clonal-level in *T*. *tabaci*, which will likely influence local variation in phenotypic traits that are commonly studied in this species, including virus transmission, host plant associations, and insecticide resistance. Our results and other recent studies demonstrate that reproductive classifications of *T*. *tabaci* are not absolute, and should not be considered fixed phenotypes that can be used to characterize different lineages of this species complex (as has been commonplace until now). Future studies aimed at characterizing the reproductive cytology and mechanisms responsible for sex determination in *T*. *tabaci* will provide insights on the influence of reproductive factors responsible for the divergence of these groups, maintenance of polyploidy, and role of limited gene flow on genetic diversity. Future studies that include genetic characterizations of the populations studied will also help characterize the number of groups present, and generate hypotheses about the ecological factors driving the large degree of phenotypic variation in traits related to reproduction, host plant preference, insecticide resistance, and virus transmission among lineages and clonal groups in *T*. *tabaci*.

### Data Archiving

Sequence data is available in Genbank: accession numbers KR152257 and KR152258

## Supporting Information

S1 Fig STRUCTURE output for K values of 2–10. (PDF)

S1 Table Collection information for NY populations of *Thrips tabaci*. (DOCX)
