# Supplementary material for: Restricted Gene Flow among Lineages of Thrips tabaci Supports Genetic Divergence Among Cryptic Species Groups
Source: PLoS One. 2016 Sep 30;11(9):e0163882. doi: 10.1371/journal.pone.0163882 (PMC5045207; doi:10.1371/journal.pone.0163882)

S1 Fig. STRUCTURE output for K values of 2-10 (K=6 in manuscript). Populations labeled on x-axis are: 1) NY-HT1 collected in 2003; 2) NY-HT1 collected in 2011-2012; 3) NY-HT2 collected in 2011-2012; 4) NY-HT3 collected in 2003; 5) NY-HT3 collected in 2011-2012; 6) NY-HT6 collected in 2011-2012; 7) NY-HA1 collected in 2003; 8) NY-HA1 collected in 2011-2012; 9) NY-HA2 collected in 2011; 10) NY-HA3 collected in 2011-2012.

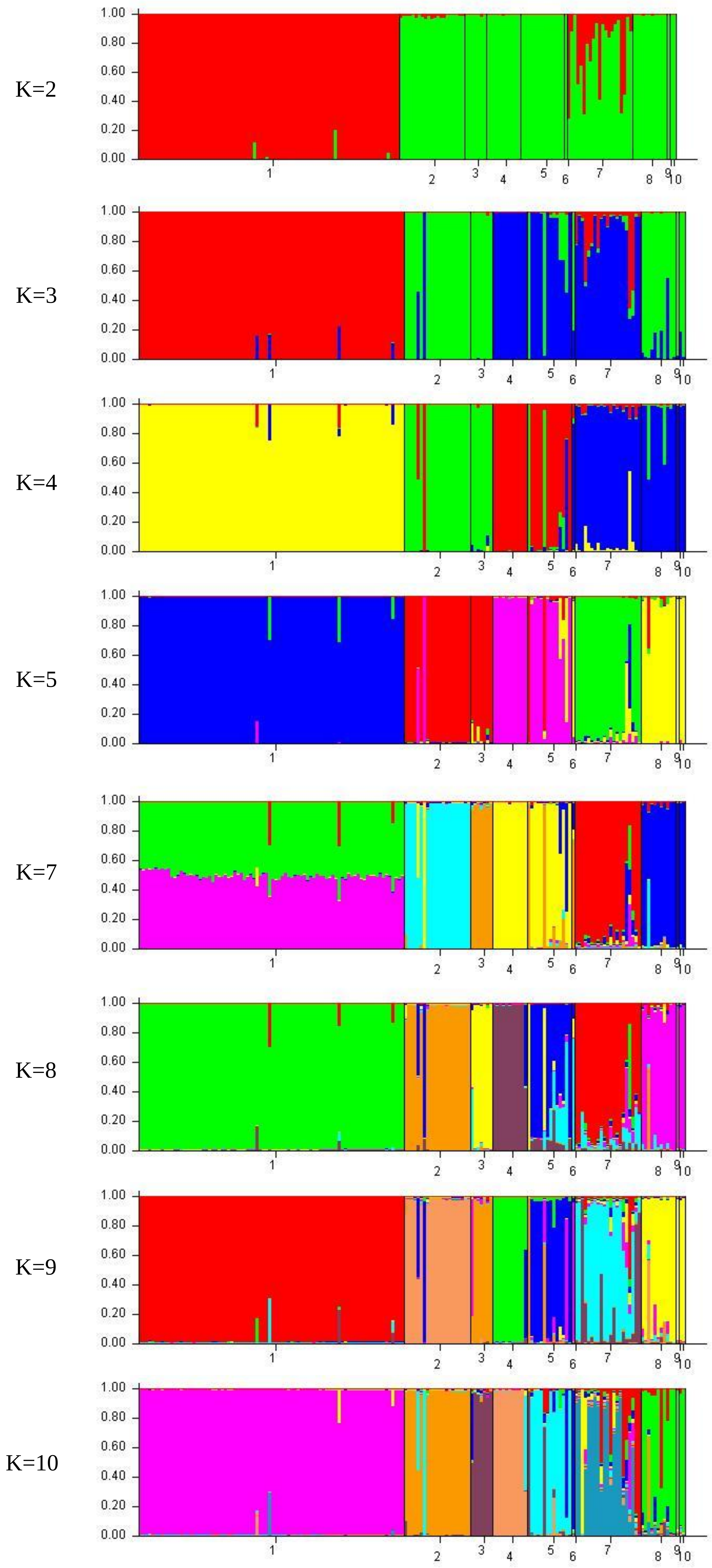

Supplement: S1 Fig — (PDF) [file pone.0163882.s001.pdf]
