# Supplementary material for: Restricted Gene Flow among Lineages of Thrips tabaci Supports Genetic Divergence Among Cryptic Species Groups
Source: PLoS One. 2016 Sep 30;11(9):e0163882. doi: 10.1371/journal.pone.0163882 (PMC5045207; doi:10.1371/journal.pone.0163882)
Supplement: S1 Table — (DOCX) [file pone.0163882.s002.docx]

**Supplemental Table 1.** Collection information for NY populations of *Thrips tabaci*.

| **County** | **COI Haplotypes** | **Field** | **Coordinates** | **Date** |
| --- | --- | --- | --- | --- |
| Orange | NY-HT1, NY-HT2, | 1  OrangeP | 41.325514, -74.419811 | 2003 |
|  | NY-HA1 | 2  OrangeR | 41.286851, -74.516070 | 2003 |
|  | NY-HT1, NY-HT2, NY-HT3, NY-HA1, NY-HA2, NY-HA3 | A | 41.346918, -74.391925 | 14Jun2012,  01Aug2012 |
|  |  | B | 41.328293, -74.398899 | 24Jun2011,  14Jun2012 |
|  |  | C | 41.324511, -74.407767 | 14Jun2012,  01Aug2012 |
|  |  | D | 41.301539, -74.482112 | 24Jun2011, 14Jun2012, 01Aug2012 |
|  |  | E | 41.293124, -74.499879 | 24Jun2011, 14Jun2012, 01Aug2012 |
|  |  | F | 41.328293, -74.398899 | 24Jun2011, 14Jun2012, 01Aug2012 |
| Oswego | NY-HT1, NY-HT2, NY-HT3, NY-HT6, NY-HA1 | 1  OSW3(6) | 43.389087, -76.565963 | 2003 |
|  |  | 2  OSW2(5) | 43.384757, -76.538401 | 2003 |
|  |  | A | 43.417674, -76.452999 | 25Jun 2011, 25Aug2011, 15Jun2012, 30Jul2012 |
|  |  | B | 43.387304, -76.536727 | 25Jun 2011, 11Jul2012, 30Jul2012 |
|  |  | C | 43.388271, -76.565223 | 25Jun 2011, 25Aug2011, |
|  |  | D | 43.405079, -76.573119 | 25Jun 2011, 25Aug2011, 15Jun2012, 30Jul2012 |
| Wayne | NY-HT1, NY-HT2,  NY-HT3, NY-HA1 | 1  WAY1(8) | 43.173223, -77.079091 | 2003 |
|  |  | 2  WAY1(7) | 43.173223, -77.079091 | 2003 |
|  |  | A | 43.17276, -77.078104 | 25Jun 2011, 25Aug2011, 15Jun2012, 30Jul2012 |
| Livingston | NY-HT1, NY-HT2, NY-HT3 | A | 42.903445, -77.945938 | 27Jun2011, 24Aug2011, 01Jun2012, 06Aug2012 |
| Genesee | NY-HT1, NY-HT2, NY-HT3 | Elba  A | 43.162994, -78.097816 | 27Jun2011, 23Aug20120, 1Jun2012, 06Aug2012 |
|  |  | Elba  B | 43.149814, -78.118372 | 27Jun2011, 23Aug2012, 01Jun2012, 01Jun2012 |
|  |  | Elba  C | 43.142205, -78.133478 | 01Jun2012, 06Aug2012 |
|  |  | Elba  D | 43.139544, -78.142104 | 27Jun2011, 23Aug2012, 01Jun2012, 06Aug2012 |
|  |  | Elba  E | 3.132936, -78.116441 | 27Jun2011, 01Jun2012, 06Aug2012 |
|  |  | Elba  F | 43.126108, -78.111334 | 27Jun2011, 23Aug2012, 01Jun2012, 06Aug2012 |
|  |  | Pembroke | 42.986442, -78.338785 | 27Jun2011, 23Aug2012, 01Jun2012, 06Aug2012 |
| Yates | NY-HT1, NY-HT2, NY-HA1 | 1  YAT2(10) | 42.701832, -77.178536 | 2003 |
|  |  | 2  YAT1(9) | 42.712846, -77.194340 | 2003 |
| Orleans | NY-HT1, NY-HT2, NY-HA1 | 1  ORL1(4) | 43.125038, -78.110602 | 2003 |
|  |  | 2  ORL2(3) | 43.132962, -78.117919 | 2003 |
